# Multi-omics investigation of thyroid development and dysfunction in down syndrome

**DOI:** 10.1093/hmg/ddag005

**Published:** 2026-02-23

**Authors:** Peter Lauffer, Nitash Zwaveling-Soonawala, Andrew Y F Li Yim, Liselot van der Laan, Shama van Zelderen-Bhola, Andrea M Venema, Adri N Mul, Marianna Bugiani, Esther Siteur-van Rijnstra, Quinn D Gunst, Maurice J B van den Hoff, Bernadette S de Bakker, Anita Boelen, Peter Henneman, A S Paul van Trotsenburg

**Affiliations:** Department of Pediatric Endocrinology, Emma Children’s Hospital, Amsterdam University Medical Center, University of Amsterdam, Meibergdreef 9, Amsterdam, AZ, 1105, Netherlands; Amsterdam Gastroenterology Endocrinology Metabolism (AGEM) Research Institute, Amsterdam University Medical Center, University of Amsterdam, Meibergdreef 9, Amsterdam, AZ, 1105, Netherlands; Department of Human Genetics, Amsterdam University Medical Center, University of Amsterdam, Meibergdreef 9, Amsterdam, AZ, 1105, Netherlands; Amsterdam Reproduction & Development Institute, Amsterdam University Medical Center, University of Amsterdam, Meibergdreef 9, Amsterdam, AZ, 1105, Netherlands; Department of Pediatric Endocrinology, Emma Children’s Hospital, Amsterdam University Medical Center, University of Amsterdam, Meibergdreef 9, Amsterdam, AZ, 1105, Netherlands; Amsterdam Gastroenterology Endocrinology Metabolism (AGEM) Research Institute, Amsterdam University Medical Center, University of Amsterdam, Meibergdreef 9, Amsterdam, AZ, 1105, Netherlands; Department of Human Genetics, Amsterdam University Medical Center, University of Amsterdam, Meibergdreef 9, Amsterdam, AZ, 1105, Netherlands; Department of Human Genetics, Amsterdam University Medical Center, University of Amsterdam, Meibergdreef 9, Amsterdam, AZ, 1105, Netherlands; Department of Human Genetics, Amsterdam University Medical Center, University of Amsterdam, Meibergdreef 9, Amsterdam, AZ, 1105, Netherlands; Department of Human Genetics, Amsterdam University Medical Center, University of Amsterdam, Meibergdreef 9, Amsterdam, AZ, 1105, Netherlands; Department of Human Genetics, Amsterdam University Medical Center, University of Amsterdam, Meibergdreef 9, Amsterdam, AZ, 1105, Netherlands; Department of Pathology, Amsterdam University Medical Center, University of Amsterdam, Meibergdreef 9, Amsterdam, AZ, 1105, Netherlands; Experimental Immunology, Amsterdam University Medical Center, University of Amsterdam, Meibergdreef 9, Amsterdam, AZ, 1105, Netherlands; Department of Medical Biology, Amsterdam University Medical Center, University of Amsterdam, Meibergdreef 9, Amsterdam, AZ, 1105, Netherlands; Department of Medical Biology, Amsterdam University Medical Center, University of Amsterdam, Meibergdreef 9, Amsterdam, AZ, 1105, Netherlands; Department of Obstetrics and Gynecology, Amsterdam University Medical Center, University of Amsterdam, Meibergdreef 9, Amsterdam, AZ, 1105, Netherlands; Amsterdam Gastroenterology Endocrinology Metabolism (AGEM) Research Institute, Amsterdam University Medical Center, University of Amsterdam, Meibergdreef 9, Amsterdam, AZ, 1105, Netherlands; Endocrine Laboratory, Department of Laboratory Medicine, Amsterdam Gastroenterology Endocrinology Metabolism (AGEM) Research Institute, Amsterdam University Medical Center, University of Amsterdam, Meibergdreef 9, Amsterdam, AZ, 1105, Netherlands; Department of Human Genetics, Amsterdam University Medical Center, University of Amsterdam, Meibergdreef 9, Amsterdam, AZ, 1105, Netherlands; Amsterdam Reproduction & Development Institute, Amsterdam University Medical Center, University of Amsterdam, Meibergdreef 9, Amsterdam, AZ, 1105, Netherlands; Department of Pediatric Endocrinology, Emma Children’s Hospital, Amsterdam University Medical Center, University of Amsterdam, Meibergdreef 9, Amsterdam, AZ, 1105, Netherlands; Amsterdam Gastroenterology Endocrinology Metabolism (AGEM) Research Institute, Amsterdam University Medical Center, University of Amsterdam, Meibergdreef 9, Amsterdam, AZ, 1105, Netherlands

**Keywords:** Down syndrome, hypothyroidism, RNA sequencing, DNA methylation, integrative methylation–expression analysis

## Abstract

**Background:**

Down syndrome (DS), caused by trisomy of chromosome 21, is associated with a high prevalence of congenital non-autoimmune thyroid dysfunction, typically characterized by an elevated serum thyroid stimulating hormone (TSH) concentration. Early-life observational studies and fetal cordocentesis data, consistently reporting elevated TSH levels, suggest a developmental origin. However, the underlying pathophysiological mechanism remains unclear. This study aimed to investigate the molecular and developmental features underlying thyroid dysfunction in DS.

**Methods:**

Thyroid tissue of fetuses with DS (n = 4) and fetuses without a genetic/developmental abnormality (n = 5) were analyzed using histology, bulk RNA sequencing (RNA-seq), and DNA methylation (DNAm) profiling.

**Results:**

Histological analysis revealed underdevelopment of DS fetal thyroid tissue, with smaller follicles and greater heterogeneity. RNA-seq identified 1035 differentially expressed genes (DEGs) distributed across the genome. Notably, three thyroid-relevant genes, *FOXE1*, *IYD*, and *DIO2*, were significantly downregulated in DS tissue. Gene set enrichment analysis (GSEA) showed widespread disruption of cellular processes. DNAm analysis identified 266 differentially methylated regions (DMRs), several of which overlapped with loci previously implicated in DS. Integration of expression and DNAm data revealed 20 significant integrative methylation–expression analysis associations, indicating cis-regulatory DNAm effects on gene expression.

**Conclusions:**

These findings suggest that congenital thyroid dysfunction in DS represents a DS-specific form of thyroid dysfunction, characterized by impaired thyroid development and altered expression and regulation of genes involved in thyroid function and general cellular processes. The genome-wide molecular changes observed likely result from gene dosage effects and systemic (epi)genomic disturbances caused by trisomy 21.

## Introduction

Down syndrome (DS) is a condition caused by trisomy of chromosome 21 [1]. It affects around one in every 750 newborns, and results in a phenotype characterized by dysmorphic features, intellectual disability, and an increased risk of several cardiovascular, gastrointestinal, respiratory, musculoskeletal and endocrine conditions. The pathophysiology of DS involves overexpression of chromosome 21 genes as well as genome-wide changes in gene expression and epigenetic regulation [1–3]. Nevertheless, the precise cause of the DS phenotype and its variability is still not fully understood.

The most common endocrine abnormalities in DS involve the thyroid gland. DS-related thyroid conditions include a higher prevalence of primary congenital hypothyroidism (CH), detected by newborn screening (NBS) [4], and a very high prevalence of (non-autoimmune) subclinical hypothyroidism (SH). In general, primary CH can be caused by thyroid maldevelopment or by thyroid hormone synthesis defects in a normally formed gland. In primary CH, serum thyroid hormone (the prohormone thyroxine, abbreviated T4) concentrations are usually low in combination with a high serum level of thyroid-stimulating hormone (the pituitary hormone controlling thyroid gland activity, abbreviated TSH). The term SH refers to more subtle thyroid dysfunction in which serum T4 levels are in the normal range, but TSH is elevated. In addition, DS individuals have a much higher tendency to develop autoimmune thyroid disease (Hashimoto’s disease) after the first years of life [5].

In DS, primary CH and non-autoimmune SH likely represent a continuum of the same group phenomenon: early in life, mean serum T4 concentrations are decreased, while mean TSH concentrations are increased [6, 7]. DS neonates with the lowest T4 and highest TSH values are detected by newborn screening and diagnosed with CH, whereas those with milder abnormalities are later diagnosed with SH. In addition, children with DS have a smaller thyroid gland than non-DS children [8]. Moreover, biochemical abnormalities are already present in prenatal stages; studies have shown elevated serum TSH and abnormal thyroid gland morphology in all studied fetuses with DS [9, 10]. Taken together, these findings suggest a shared, underlying mechanism that affects early thyroid development or function in most individuals with DS, independent of rare, individual-specific genetic variants. While a single case report has described a *PAX8* promoter variant in a child with DS and primary CH (*PAX8* is a gene implicated in monogenic thyroid maldevelopment), such findings are rare and not thought to explain the high prevalence of thyroid dysfunction observed in DS at the population level [11].

This study aimed to investigate the molecular and developmental features underlying the spectrum of non-autoimmune thyroid dysfunction observed in DS. We hypothesized that trisomy of chromosome 21 leads to unique genome-wide changes in gene expression and epigenetic regulation in the thyroid gland. To accomplish our goal we studied histological sections, analyzed high-throughput bulk RNA sequencing (RNA-seq) data and DNA methylation (DNAm) data of human fetal thyroid tissue. Functional integrative effects of altered DNAm on gene expression were studied in an integrative methylation–expression analysis. The results of this study shed new light on the molecular abnormalities that occur in the fetal DS thyroid gland.

## Results

Fetal thyroid tissues from six fetuses with DS and six healthy fetuses were analyzed using histology, RNA sequencing (RNA-seq), and DNA methylation (DNAm) profiling. Due to sample quality and assay-specific constraints, not all samples were included in every analysis (Supplementary Fig. 1, Supplementary Fig. 2). The final number of samples used per modality is detailed in the Methods and Supplementary Table 1.

### Down syndrome fetal thyroid tissue shows underdevelopment compared to healthy control tissue

**Figure 1:**
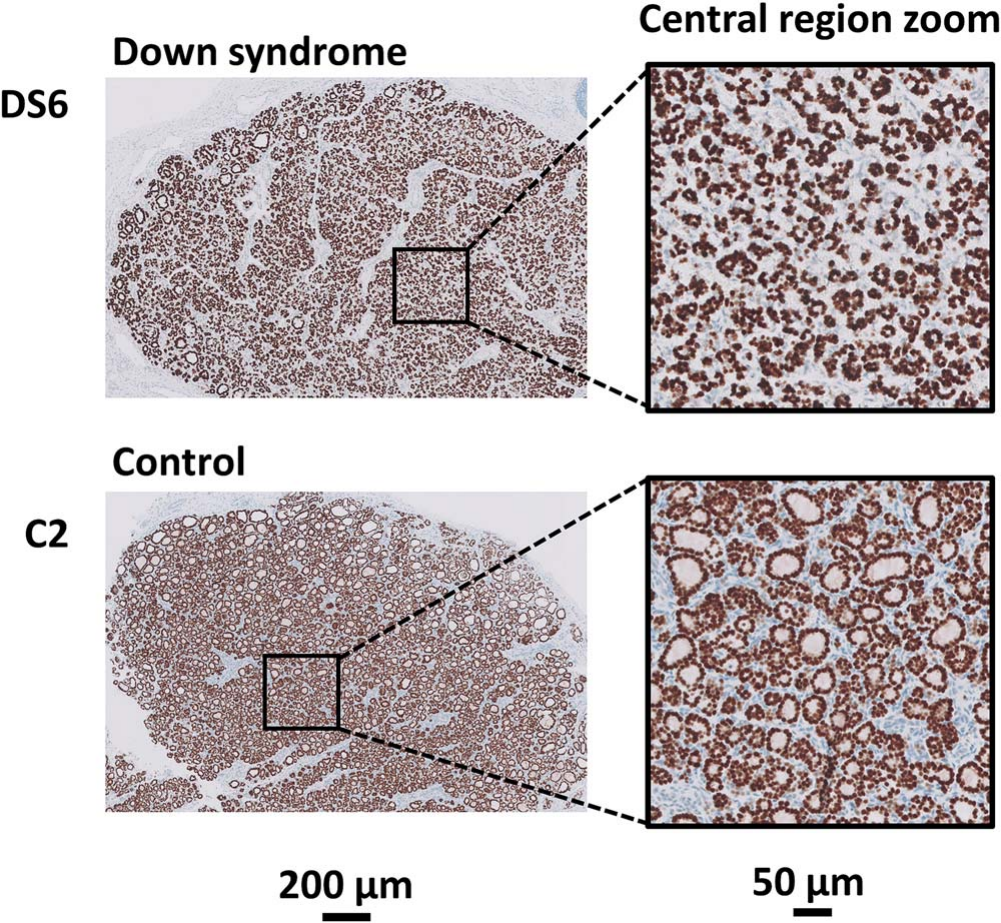
Histological findings in DS fetal thyroid tissue. Comparison of DS fetal thyroid tissue of sample DS6 with fetal thyroid tissue of control sample C2 (gestational age 17 to 20 weeks, when the thyroid gland reaches structural maturity). Immunolabeling of the images shown was performed with TTF1. Of both sections, a zoom-in of the central region is given, showing smaller follicle size in the central region in DS fetal thyroid tissue compared to non-DS/healthy thyroid tissue. This analysis focuses on a direct comparison of the most visually observable difference, therefore only two samples were selected for this specific observation. All sections are shown in Supplementary Fig. 3.

To assess structural differences in thyroid development between DS and control fetuses, we performed histological evaluation of paraffin-embedded thyroid tissue sections stained for the thyroid-specific transcription factor NKX2–1 (TTF1). In DS samples, follicles in the central region appeared consistently smaller than in controls, a sign of underdevelopment or lagging maturation (Fig. 1, Supplementary Fig. 3) [12]. The range of follicle sizes was also broader in DS tissue, leading to a more heterogeneous architecture, rather than the typical gradient of increasing follicle size from center to periphery. While these findings support the notion of impaired thyroid development in DS [10], it should be noted that the observations were qualitative, as no formal morphometric analysis was performed.

**Figure 2:**
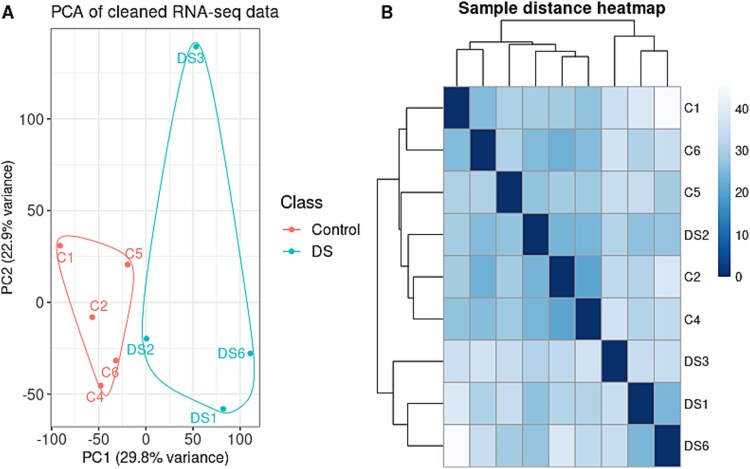
Principal component analysis (PCA) and sample distance heatmap of bulk RNA-seq data. (A) PCA results of the first and second PCs plotted, visualizing the RNA-seq profiles of the study cohort. The sex confounder was removed from the data prior to analysis by residualizing log_2_-transformed counts data. Samples clustered according to condition, and groups were noticeably separated in PC1. (B) Sample distance heatmap showing the hierarchical clustering of DS samples and age-matched controls. Each row and column represents one sample. Colors in cells represent pairwise distances among samples, calculated with classical multidimensional scaling.

**Figure 3:**
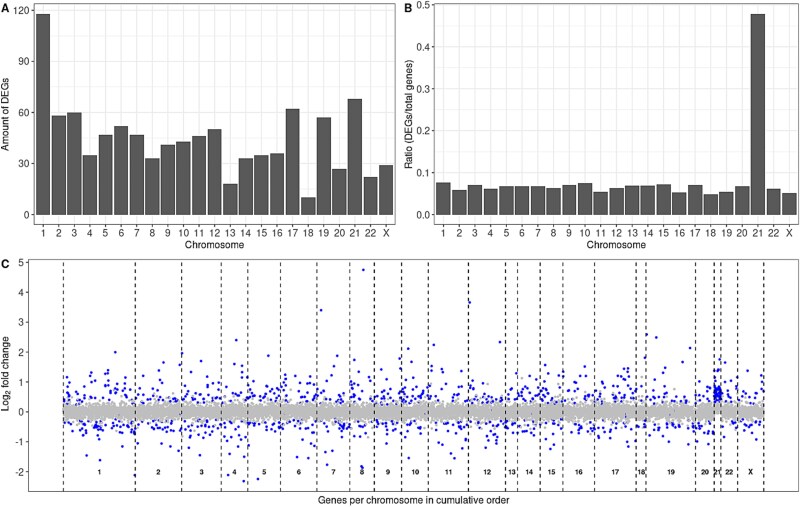
Differential gene expression analysis results. (A) Differentially expressed genes (DEGs) per chromosome. (B) DEGs per chromosome divided by the total amount of expressed genes per chromosome. DEGs are clearly enriched for chromosome 21. (C) Genes in cumulative order per chromosome visualized, including log2 fold change in expression. DEGs are indicated with a blue dot, while non-significant genes are indicated with grey dots. Notably, chromosome 21 DEGs are all overexpressed in DS fetal thyroid tissue compared to controls.

### RNA sequencing analysis shows differentially expressed genes in down syndrome fetal thyroid tissue

To investigate the possible molecular defects underlying congenital thyroid dysfunction in DS, and the observed histological findings, we analyzed bulk RNA-seq data from fetal thyroid tissue. After quality control (Methods), PCA identified distinct separation between DS and control samples (Fig. 2A), with one sample (DS2) clustering with controls (Fig. 2B), reflecting known heterogeneity in DS. Differential expression analysis revealed 1035 differentially expressed genes (DEGs) with false discovery rate (FDR)-adjusted *P*-value < 0.05 (Supplementary Table 2), of which 589 were upregulated, and 446 downregulated (Fig. 3). As expected in the context of a dosage effect, DEGs were enriched for chromosome 21 (*P*-value < 0.05; Fig. 3). All chromosome 21 DEGs were overexpressed. DEGs were uniformly distributed across all chromosomes if chromosome 21 was not taken into account (*P*-value = 0.25), with approximately 7% of expressed genes on each chromosome showing differential expression between DS and controls. Also, no significant overrepresentation of either upregulated or downregulated DEGs was observed for the rest of the chromosomes (*P*-value = 0.58).

Of the 145 expressed genes mapped and quantified for chromosome 21, 68 genes (47%) were significantly overexpressed in DS fetal thyroid tissue (Supplementary Table 2). Mean absolute fold change of these genes was 1.57 (range 1.25–3.37), as expected from a 50% increase in expression.

Looking specifically at genes implicated in thyroid function in non-DS newborns and young children [13], we identified three DEGs that were significantly downregulated in DS fetal thyroid tissue: *FOXE1*, *IYD* and *DIO2* (Supplementary Table 3, Supplementary Fig. 4). All three genes ranked among the top 200 DEGs in the cohort based on statistical significance (Supplementary Table 3). *FOXE1* encodes a transcription factor essential for thyrocyte migration and survival, *IYD* is involved in iodide recycling in the thyroid gland, and *DIO2* catalyzes the conversion of thyroxine (T4) to its active form triiodothyronine (T3) [14–16].

### Gene set enrichment analysis suggests upregulation and downregulation of various processes vital for normal cell function in down syndrome fetal thyroid tissue

**Figure 4:**
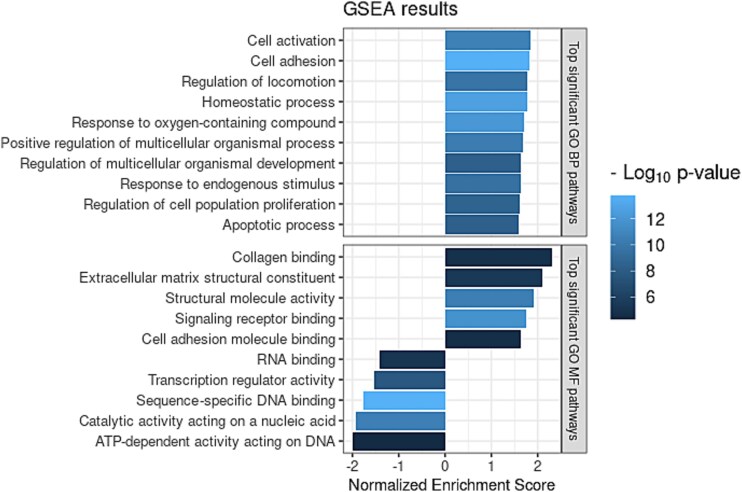
Top gene set enrichment analysis (GSEA) results. Representation of the leading GSEA outcomes. Panels display the top ten significant gene sets of gene ontology (GO) biological process (BP) gene sets and GO metabolic function (MF) gene sets.

To identify significantly enriched gene sets among DEGs in DS thyroid tissue, we conducted gene set enrichment analysis (GSEA). 155 gene ontology (GO) biological processes and 39 GO molecular functions were significantly enriched (FDR-adjusted *P*-value < 0.01) (Supplementary Table 4). The top significant and enriched gene sets were associated with a wide array of cellular processes and functions (Fig. 4). A number of gene sets including ion homeostasis and extracellular matrix organization had a positive normalized enrichment score (NES), meaning that genes associated with these gene sets were more prevalent at the top of the ranked gene list (most upregulated significant genes), whereas gene sets mainly associated with nuclear processes had a negative NES (most downregulated significant genes) (Supplementary Table 4).

To further explore the functional implications of DEGs, we performed a similarity-based network analysis of enriched gene sets, revealing eight clusters of significantly enriched gene sets (Supplementary Fig. 5). Two clusters were composed of downregulated gene sets related to nuclear processes such as cell cycle regulation and DNA repair. The remaining six clusters consisted of upregulated gene sets, including pathways involved in immune response, cellular and tissue development, signal transduction, enzyme regulation, and ion transport.

### Genome-wide DNA methylation analysis identifies differentially methylated regions in down syndrome fetal thyroid tissue

**Figure 5:**
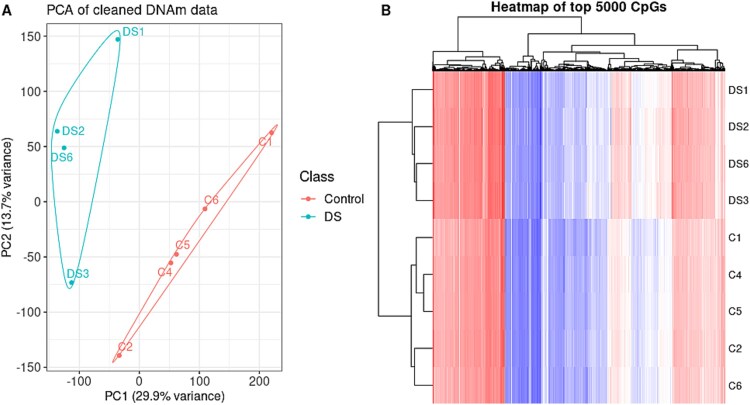
Principal component analysis (PCA) and CpG heatmap of DNAm data. (A) PCA results of the first and second PCs plotted, visualizing the DNAm profiles of the study cohort. Sex confounder and a surrogate batch effect were removed from the data prior to analysis by residualizing β-values data. Samples clustered according to condition, and groups were noticeably separated in PC1. (B) Heatmap showing the hierarchical clustering (and separation) of DS samples and age-matched controls, based on DNAm of the top 5000 CpGs. Each row represents a sample, and each column represents a CpG. Colors in cells represent β, ranging from fully methylated (red), to unmethylated (blue).

As epigenetics could underlie potential differences in expression, we next interrogated the DNA methylome using the Illumina Infinium MethylationEPIC BeadChip array. PCA of DNAm data for data exploration showed clear segregation of DS thyroid tissue and control thyroid tissue (Fig. 5A). Considering the top 5000 CpGs from DNAm analysis, we observed complete separation of the two groups (Fig. 5B). Subsequent differential DNAm analysis revealed three differentially methylated positions (DMPs; CpGs with a significant methylation index (β) difference between DS and control samples) associated with DS (Supplementary Table 5). The three DMPs were hypomethylated in DS, and mapped to the gene bodies of *JAK2* and *CPN8,* and to the region encoding the 5’UTR of *APBB1.* Investigation of genes implicated in thyroid function in non-DS newborns and young children (by selecting CpGs that map within 2000 bp range of thyroid-related genes) [13], yielded no statistically significant DMPs. Through differentially methylated region analysis (DMR; a consecutive stretch of CpGs where at least two presented a mean difference in β of 0.05 at a FDR-adjusted *P*-value < 0.01), we identified 266 epigenome-wide DMRs, that could be annotated to 331 genes (Supplementary Table 6). One hypermethylated DMR was annotated to *CDCA8*, a thyroid-related gene. However, *CDCA8* did not present DS-associated difference in expression (*P*-value 0.19, FDR 0.49). Among the 266 DMRs, several overlapped with loci previously reported as differentially methylated in DS across other tissues. Notably, we identified a hypermethylated DMR in *CPT1B*, a hypomethylated DMR in *ADNP2*, and hypermethylated DMRs in *PCDHGA5* (Supplementary Table 6). These DMRs have previously also been reported in DS fetal cortex, adult heart, placenta, and blood [3, 17–23].

### Identification of expression quantitative trait methylation probes in down syndrome fetal thyroid tissue

**Figure 6:**
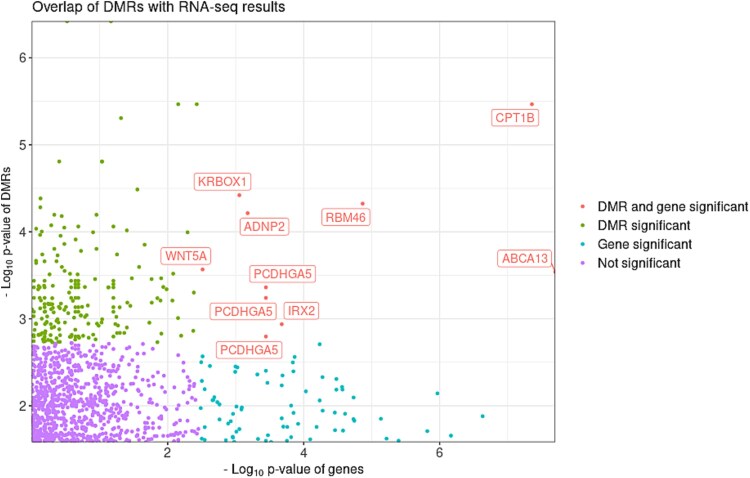
Overlap of bulk RNA-seq and DNA methylation (DNAm) results. Intersection between RNA-seq and DNAm findings. X-axis represents RNA-seq analysis p-values, and y-axis represents p-values of DMRs (annotated to the genes). Names of DEGs with significant DMRs are given.

To investigate cis-regulatory DNAm elements that regulate the transcription of DS-associated DEGs, we investigated whether DMRs occurred within < 2000 bps (upstream and downstream) of the DEGs. Altogether, we identified ten potential DMR-DEG pairs (Table 1; Fig. 6; Supplementary Table 7). We subsequently performed integrative methylation–expression analyses to correlate DNAm and gene expression. In total, 20 significant integrative methylation–expression analysis associations were identified, encompassing 129 CpG sites (Supplementary Table 8; Supplementary Fig. 6). Of these, 18 showed a negative correlation between methylation and gene expression (i.e. hypomethylation associated with higher expression), while two showed a positive correlation.

**Table 1:** DMRs annotated to a DEG.

Gene	DMR (hg19)	FDR DMR	Delta β DMR	FDR expression	Log_2_ fold change expression	Annotation (hg19)
IRX2	chr5:2753876–2 754 148	8.32E-03	−0.11	8.11E-03	−1.12	Promoter
KRBOX1	chr3:42977643–42 978 318	3.69E-03	0.12	2.08E-02	−0.85	Promoter
ABCA13	chr7:48494362–48 495 072	5.93E-03	0.14	1.01E-05	−1.77	Promoter
CPT1B	chr22:51016386–51 017 162	1.22E-03	0.18	1.81E-05	1.15	Last exon
WNT5A	chr3:55517101–55 518 131	5.85E-03	0.09	4.72E-02	−0.32	Promoter
ADNP2	chr18:77905119–77 905 947	4.13E-03	−0.15	1.75E-02	−0.30	Promoter
PCDHGA5	chr5:140744763–140 744 983	9.31E-03	0.15	1.17E-02	0.70	Promoter
PCDHGA5	chr5:140748559–140 749 358	7.25E-03	0.19	1.17E-02	0.70	Gene body
PCDHGA5	chr5:140760983–140 761 737	6.27E-03	0.17	1.17E-02	0.70	Gene body
RBM46	chr4:155702341–155 703 170	3.98E-03	0.10	1.20E-03	−2.31	Promoter

## Discussion

Our study investigated the impact of an extra chromosome 21 on fetal thyroid development by analyzing histological sections, bulk RNA-seq, and DNAm profiles from four DS and five control fetal thyroid tissues.

To explore group-level transcriptional effects of trisomy 21 in the fetal thyroid, we performed a DEG analysis of bulk RNA-seq data, where we notably identified downregulation of *FOXE1*, *IYD*, and *DIO2*, genes linked to embryonic thyroid development or thyroid function. We hypothesize that this downregulation contributes to impaired thyroid function in DS through three mechanisms: [1] reduced *FOXE1* activity may impair thyroid morphogenesis and follicular maturation; [2] decreased *IYD* expression could limit iodide recycling, reducing thyroid hormone synthesis; and [3] diminished *DIO2* expression may lower local T3 availability, potentially affecting both thyroid development and intracellular metabolic regulation. While pathogenic variants in *FOXE1* and *IYD* cause rare monogenic hypothyroidism, their consistent downregulation at the group level in DS suggests a shared dosage-sensitive regulatory disruption, rather than isolated pathogenic variants.

In addition to thyroid-specific genes, we identified more than 1000 DEGs distributed across all chromosomes, and gene ontology analysis revealed enrichment of fundamental biological processes. For example, pathways related to cell adhesion, homeostatic and metabolic processes, responses to endogenous stimuli, as well as cell proliferation and apoptosis, were all significantly disrupted in DS thyroid. These findings are consistent with transcriptional disturbances described in other DS tissues [24, 25]. The observed cross-tissue patterns of gene dysregulation raise the possibility that aspects of thyroid dysfunction in DS may stem from systemic effects of trisomy 21, rather than thyroid-specific mechanisms alone. Supporting this notion, several DMRs identified in our study, such as those in *CPT1B*, *ADNP2*, and *PCDHGA5*, have also been observed in a range of DS tissues, including fetal cortex, adult heart, placenta, and blood [17–19, 21, 23]. This recurrence suggests that certain epigenetic alterations originate early in embryonic development and persist across lineages, echoing a sustained, cross-tissue molecular impact of trisomy 21. Further investigation of this cross-tissue molecular signature of DS is needed to determine its role in DS pathophysiology and disease severity.

A recently published preprint manuscript on a single-cell experiment in fetal DS thyroid gland also reported genome-wide transcriptomic changes [26]. While methodological differences limit direct comparison with our study, we do observe considerable overlap on a gene-set level. Results regarding individual thyroid-related genes do not overlap, which could possibly be explained by the studied gestational ages (gestational age between 11 and 17 weeks vs approximately 18 weeks in our study).

One interesting observation from the GSEA is the upregulation of immune-related gene sets. Although the thyroid dysfunction in the studied DS cases was non-autoimmune, individuals with DS have a well-known increased risk of developing Hashimoto’s thyroiditis later in life. The immune signal we observe may therefore reflect an underlying susceptibility.

To explore whether the observed epigenetic alterations may contribute directly to transcriptional dysregulation, we performed an integrative methylation–expression analysis. This revealed 20 promoter-associated methylation changes that correlated with decreased expression of genes in DS thyroid tissue, including seven hypermethylated DMRs annotated to the chromosome 5 clustered protocadherin locus (harboring genes implicated in neuronal network development), associated with decreased expression of several protocadherin genes, which accords with earlier observations in DS fetal brain [19, 27]. The other 13 identified methylation-expression associations are novel functional DNAm-gene expression associations in the context of DS. One of these, the *ADNP2* methylation-expression association, is characterized by a hypomethylated DMR annotated to the last exon, and decreased expression of *ADNP2*, which encodes activity-dependent neuroprotective protein 2 (ADNP2). Notably, *ADNP2* was previously found in DS-related epigenetic studies of other tissues [21, 22]. *ADNP2* has been implicated in oxidative stress response, with experimental silencing shown to increase susceptibility to cell death [28]. Therefore, we hypothesize that *ADNP2* dysregulation could play a role in exacerbating cellular dysfunction in DS, already in early stages of development, and across tissues.

In previous work on DS epigenetics, we identified 31 thyroid-related DMPs in a meta-analysis of DNAm profiles of DS postnatal blood samples [29]. These DMPs were mostly hypomethylated, and promoter associated, suggesting upregulation of thyroid genes. We hypothesized an epigenetic ‘rescue phenomenon,’ at which DNAm of thyroid-related genes was altered in an attempt to ameliorate thyroid status, by influencing gene expression. In contrast to earlier findings, no evidence of altered DNAm (also considering DMRs) was detected in the previously identified hypomethylated thyroid genes [29]. According to the data of the current study, we cannot confirm that a possible DNAm rescue phenomenon plays a role in DS fetal thyroid tissue.

Our work presents several limitations. First, the small number of available fetal samples limited statistical power. For example, about 850 000 features were profiled in the genome-wide DNAm analysis, and the imbalance between features tested and the small sample size, likely reduced sensitivity to detect DMPs.

Second, we acknowledge that in our bulk multi-omics analysis, we may have also analyzed signals stemming from other cells than thyrocytes. To reduce the chance of including samples with little thyroid tissue, we used a two-step filtering approach, first checking thyroid- and skeletal muscle–specific gene expression and then robust PCA on the remaining samples. Cell-type proportion estimates could not be included, as no reference panel is available for age-matched human fetal thyroid tissue. In addition, reference-based deconvolution approaches are expected to perform poorly in DS samples due to the transcriptomic effects of trisomy 21. We therefore relied on our two-step filtering strategy to reduce the impact of cellular heterogeneity and to avoid inclusion of samples with low thyrocyte content.

Third, the unavailability of thyroid phenotype/outcome data precluded sample selection based on these criteria. Thus, our analysis assumes a DS thyroid dysfunction versus normal function in controls, supported by consistently observed elevated serum TSH in DS fetuses [9, 10]. This limitation underscores the need for further research to explore DS phenotypic variability, potentially through integrating multi-omics data with thyroid hormone measurements.

Lastly, histological analysis was performed using a single marker (TTF1/NKX2–1), which allowed us to corroborate earlier observations of fetal thyroid abnormalities in DS [10]. Further insight could likely be obtained by extending the analysis to additional markers of thyroid maturation, such as thyroglobulin (TG), thyroid peroxidase (TPO) and the thyrotropin receptor (TSHR). In addition, focus on the thyroid-related DEGs identified in our study (*FOXE1*, *IYD* and *DIO2*), may be informative targets for future histological work.

Collectively, our findings suggest that congenital thyroid dysfunction in DS represents a DS-specific developmental disorder of the thyroid. The (epi)genome-wide disturbances we observed likely reflect both direct dosage effects of chromosome 21 genes and broader trisomy-induced genomic instability. Some affected pathways appear cross-tissue, highlighting potential targets for future mechanistic and pharmacological studies.

## Materials and methods

### Samples

DS and non-DS/healthy thyroid tissue was isolated within eight hours after fetal death, following elective induced abortion. Pregnancies of non-DS/healthy were aborted for non-medical reasons (social abortion), with no known genetic or structural abnormalities, based on prenatal ultrasound and macroscopic postmortem examination. DS was diagnosed by non-invasive prenatal testing. Gestational age of fetuses was determined by measurement of crown-rump length by ultrasound around 12 weeks gestational age. Samples were obtained from the Dutch Fetal Biobank (Amsterdam University Medical Center, University of Amsterdam), HIS Mouse Facility (Amsterdam University Medical Center, University of Amsterdam) and Bloemenhove clinic (Heemstede, Netherlands). Right thyroid lobe samples were fixed overnight in 4% (w/v) paraformaldehyde (PFA) freshly dissolved in PBS (10 mM NaH_2_PO_4_/Na_2_HPO_4_, 150 mM NaCl, pH 7.6), and stored in 70% ethanol at 4°C until histological evaluation. Left thyroid lobe samples were stored in RNAlater (Thermo Fisher Scientific, Waltham, MA, USA) and kept frozen at −80°C until RNA and DNA isolation.

In total, thyroid tissue of 12 fetuses, was obtained from donations after elective termination of pregnancy. Gestational age of fetuses with DS was 18.3 ± 0.7 weeks, and of controls 18.6 ± 0.2 weeks. Five right thyroid lobes (two of a fetus with DS) were fixed in paraformaldehyde for histological evaluation, while 12 left thyroid lobes (six of a fetus with DS) were stored in RNAlater for RNA-seq and DNAm profiling (Supplementary Table 1). Sex and the presence of an extra chromosome 21 were confirmed by quantitative fluorescent polymerase chain reaction (QF-PCR) (Supplementary Table 1).

### Histological evaluation

Samples were formalin fixed and embedded in paraffin in the transverse plane and stained with H&E and TTF1 (NKX2–1) for histological analysis.

### RNA library preparation, sequencing and data analysis

Total RNA was isolated from left lobe from thyroid samples using a hand-held homogenizer and the Promega ReliaPrep RNA Miniprep System (Thermo Fisher Scientific). RNA yield was determined with the NanoDrop Microvolume Spectrophotometer (Thermo Fisher Scientific). RIN values were obtained using a Fragment analyzer 5200 (Agilent Technologies, Santa Clara, CA, USA). Fragmentation and mRNA library preparation was performed using the Kapa mRNA Hyperprep Kit (Roche, Basel, Switzerland). Libraries were equimolar pooled and quality was checked on a TapeStation system using the DNA1000 ScreenTape (Agilent Technologies). Libraries were sequenced with poly(A) selection to sequence all messenger RNA for gene expression analysis on the NovaSeq6000 PE150 (Illumina, San Diego, CA, USA), producing at least 40 M 150-bp paired-end reads per library.

Quality profiling of raw data was performed using FastQC (v0.11.9) and fastp (v0.22.0) [30, 31]. Quality reports were summarized with MultiQC (v1.13) [32]. Reads were trimmed for adapter sequences and poor reads were filtered with fastp (v0.22.0) employing the default parameters [30]. Trimmed reads were mapped to the GENCODE human (protein coding) transcriptome (v42) and quantified with Salmon (v1.9.0), correcting for fragment GC content bias [33]. Transcript-level quantification data was imported in R (v4.2.1) with tximeta (v1.16.0) and summarized to the gene-level [34].

Isolated RNA of all left thyroid lobe samples was of sufficient quality (RNA integrity number > 7). Between 41.5 and 80.5 million reads were generated for each sample, which were mapped to the human reference transcriptome and quantified. Mapping rates were between 66.8% and 82.5%. After filtering steps, selecting for expressed genes, 15 556 genes remained for analysis, approximately 75% of possible genes (according to the GENCODE human [protein coding] transcriptome).

For additional quality profiling of processed data, we employed the PCAgrid algorithm from rrcov (v1.7–2) to perform robust Principal Component Analysis (rPCA), with the aim of sample outlier detection [35]. The PCAgrid algorithm has been shown to be an accurate method for RNA-seq data with small sample sizes [36]. Sex-related confounder effects were removed from processed data with the removeBatchEffect function of limma (v3.46.0) to visualize cleaned data with a classical PCA and sample distance heatmap [37].

Based on the expression levels of thyroid marker genes *TPO*, *TG*, *TSHR*, and *SLC5A5*, two DS samples did not seem to be derived from the thyroid gland but likely originated from skeletal muscle, based on PCA and the expression of skeletal muscle marker genes *MYH1* and *MYOD1* (Supplementary Fig. 1). To further explore data quality (after removal of abovementioned samples), we performed rPCA, which identified one statistically deviant control sample (Supplementary Fig. 2). These samples were removed from the dataset before differential expression analysis.

Differential expression of genes was investigated with DESeq2 (v1.36.0) [38]. As an initial filtering step, genes were filtered from the data if there were less than ten counts in four samples. Afterwards, DESeq2 performed independent filtering using the mean of normalized counts as a filter statistic. For detection of differentially expressed genes (DEGs), we used the DESeq2 generalized linear model, with condition (DS or control) as the independent variable, and gene-specific counts as the dependent variable. Sample sex was added as a covariate in the model formula. Genes with an Benjamini-Hochberg FDR-adjusted *P*-value < 0.05 were considered significant. To reduce noise from low counts and/or highly variable counts across samples, and better estimate effect sizes for gene clustering and ranking, we used the Approximate Posterior Estimation for generalized linear model (apeglm) method to shrink logarithmic fold changes (LFC) [39]. Enrichment of (upregulated or downregulated) DEGs for specific chromosomes was tested with a chi-squared test.

Three samples (two DS, one control) were not fit for differentially expressed gene (DEG) and DNAm analysis and removed from the dataset (Supplementary Fig. 1, Supplementary Fig. 2).

### DNA library preparation, processing and analysis

DNA was isolated from thyroid samples using the Maxwell RSC Tissue DNA Kit #AS1610 (Promega Corportation, Madison, WI, USA) according to manufacturer’s guidelines. DNA quantity, quality control and DNAm profiling was performed by the Core Facility Genomics (Amsterdam University Medical Center, University of Amsterdam, Amsterdam, Netherlands) (ISO/IEC 17025 certified). In short, bisulfite converted DNA was amplified and subsequently hybridized on the EPIC (HM850K) array, according to the manufacturer’s protocol.

Raw data quality control was done with MethylAid (v1.30; default settings) [40]. DNAm data of all samples passed MethylAid quality control checks. Allosomal probes, chromosome 21 probes, probes known to involve genetically polymorphic sites (minor allele frequency > 0.01), or probes susceptible to cross-hybridization were removed from the dataset. Next, data were normalized using the preprocessFunnorm function of minfi (v.1.42) [41]. PCA was performed in order to explore the data with the prcomp function. DMPs were detected using minfi (v1.42), applying linear model function limma (lmfit, v3.52.4) package [37, 41], with condition (DS or non-DS) as the independent variable and CpG-specific normalized M-values as the dependent variable. Sex and surrogate batch effects—calculated with a surrogate variable analysis [42], were included in the analysis as covariates. DMPs with an FDR-adjusted p-value < 0.05 were considered as genome-wide significant.

DMRs were detected using bumphunter (v1.42.0). DMRs needed to consist of at least three CpGs. The β area cut-off was set at 0.05. To calculate *P*-values, a DMR null-distribution was computed based on 100 iterations of sample permutation, which was compared to the actual distribution of DMRs. The Benjamini-Hochberg procedure was used to control the FDR. DMRs with FDR-adjusted *P*-value < 0.01 were considered significant.

### Quantitative fluorescent polymerase chain reaction

QF-PCR analysis was performed using the QST*Rplusv2 kit of Elucigene (Manchester, UK) according to the manufacturer’s guidelines. Interpretation of data was performed according standard and accredited procedures of the Amsterdam UMC, Human Genetics Department.

### Integrative methylation–expression analysis

Methylation-expression associations were calculated as previously described [43]. To summarize: in each sample, the median level of methylation was determined for each DMR (DMRs with FDR-adjusted *P*-value < 0.01 were selected for this analysis). Then, Pearson's correlation coefficients were calculated for the median methylation levels of the DMRs and the log-transformed counts of the corresponding genes. To estimate the confidence intervals with 95% certainty, a bootstrapping method using 100 000 resamples was employed. *P*-values were obtained by comparing the correlation coefficients with a null distribution obtained through resampling. DMR-gene correlations with *P*-values < 0.05 were deemed statistically significant.

### Gene set enrichment analysis

Overrepresentation analysis and GSEA were performed with gene sets downloaded from MSigDB (https://www.gsea-msigdb.org/gsea/msigdb/index.jsp) [44], using the overrepresentation and gene set test procedures of fgsea (v1.24.0) [45]. Gene sets with an FDR-adjusted *P*-value < 0.01 were considered significant. Gene sets with overlapping genes were collapsed into parents gene sets. The vissE package (v1.8.0) was used to interpret and further visualize GSEA results [46], employing the Jaccard index (threshold = 0.25) to compute clusters of overlapping gene sets.

## Supplementary Material

SupplementalFig1_ddag005

SupplementalFig2_ddag005

SupplementalFig3_ddag005

SupplementalFig4_ddag005

SupplementalFig5_ddag005

SupplementalFig6_ddag005

SupplementalTable1_ddag005

SupplementalTable2_ddag005

SupplementalTable3_ddag005

SupplementalTable4_ddag005

SupplementalTable5_ddag005

SupplementalTable6_ddag005

SupplementalTable7_ddag005

SupplementalTable8_ddag005

LaufferSupplementaryData_ddag005

## Data Availability

Summary statistics supporting the results in this manuscript have been provided as Supplementary Tables. Raw gene expression sequencing data (.fastq) and raw DNA-methylation profiles (.idat) and metadata of the results presented in this manuscript are available on reasonable request and under a data transfer agreement (DTA) following the Dutch data protection act (DPA) at the European Genome–Phenome Archive: https://ega-archive.org/studies, under accession identifier EGAS00001007677. Further information about EGA can be found on https://ega-archive.org ‘The European Genome-phenome Archive of human data consented for biomedical research’ (http://www.nature.com/ng/journal/v47/n7/full/ng.3312.html).
