# Supplementary figures and images for: Multi-omics investigation of thyroid development and dysfunction in down syndrome
Source: Hum Mol Genet. 2026 Feb 23;35(4):ddag005. doi: 10.1093/hmg/ddag005 (PMC13036834; doi:10.1093/hmg/ddag005)

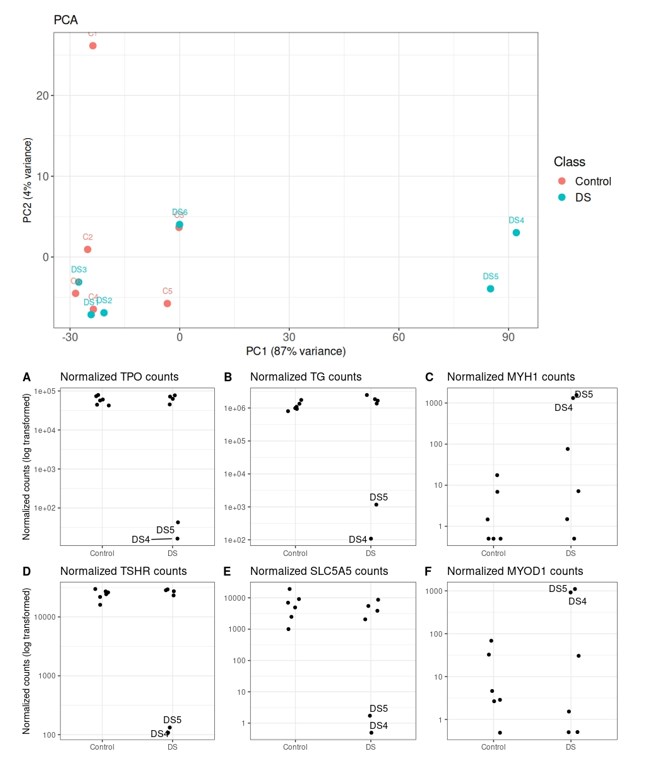

Supplement: SupplementalFig1_ddag005 [file supplementalfig1_ddag005.jpeg]

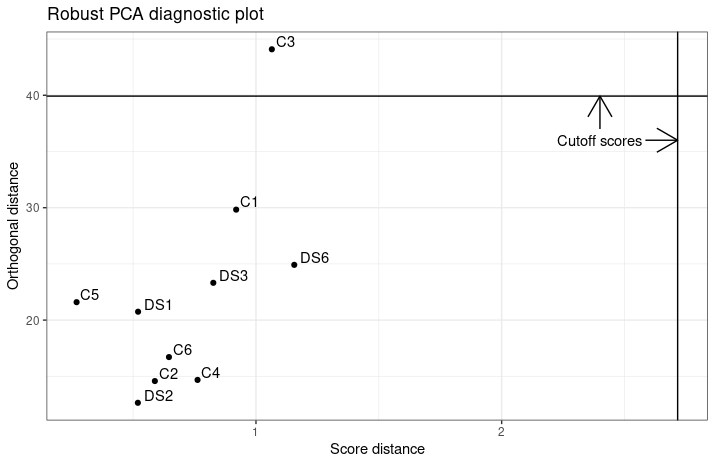

Supplement: SupplementalFig2_ddag005 [file supplementalfig2_ddag005.jpeg]

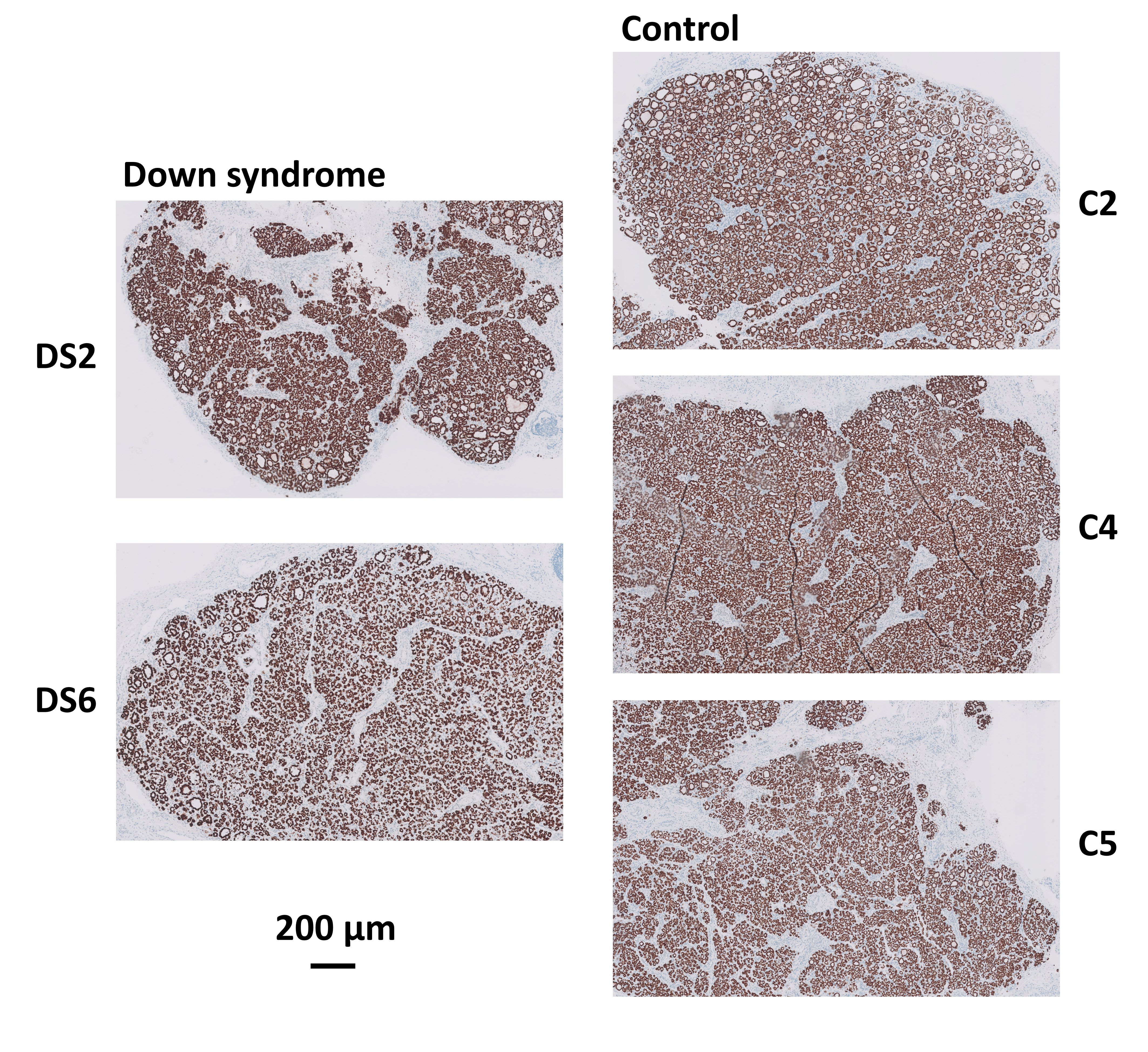

Supplement: SupplementalFig3_ddag005 [file supplementalfig3_ddag005.jpeg]

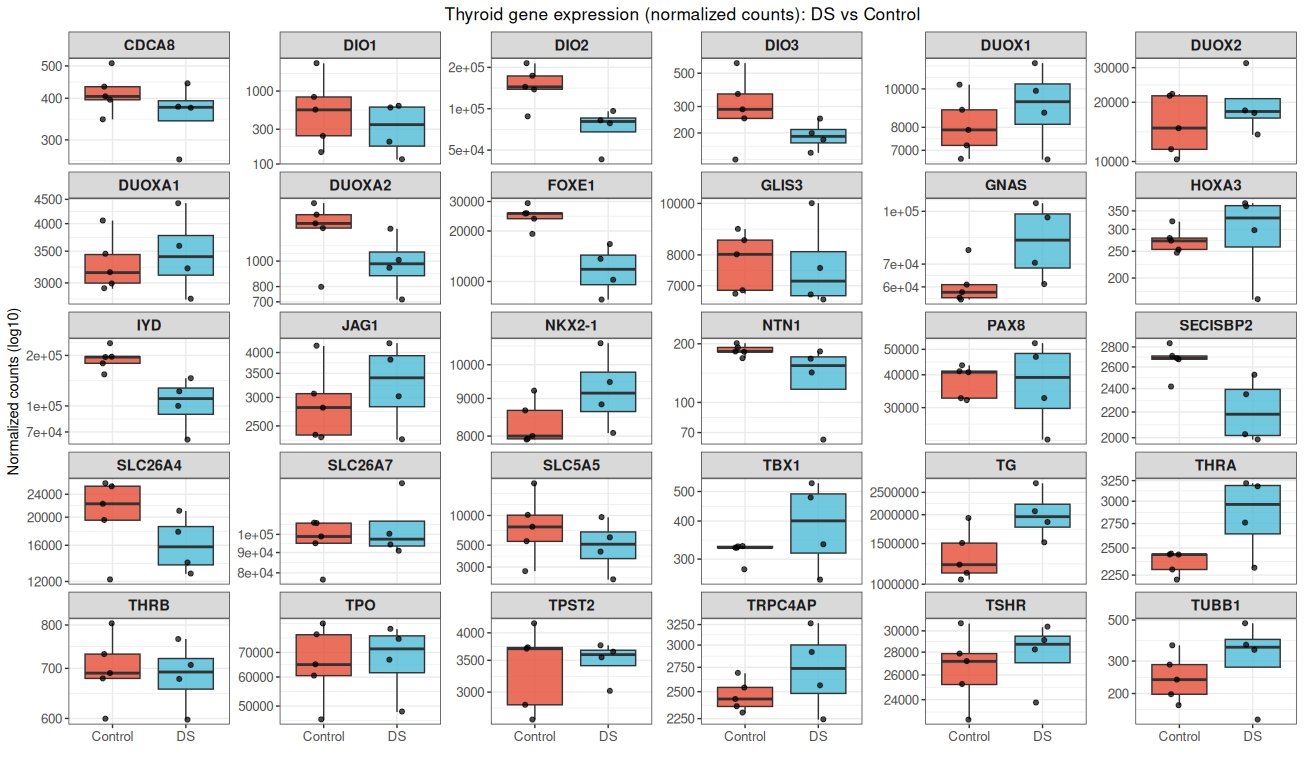

Supplement: SupplementalFig4_ddag005 [file supplementalfig4_ddag005.jpeg]

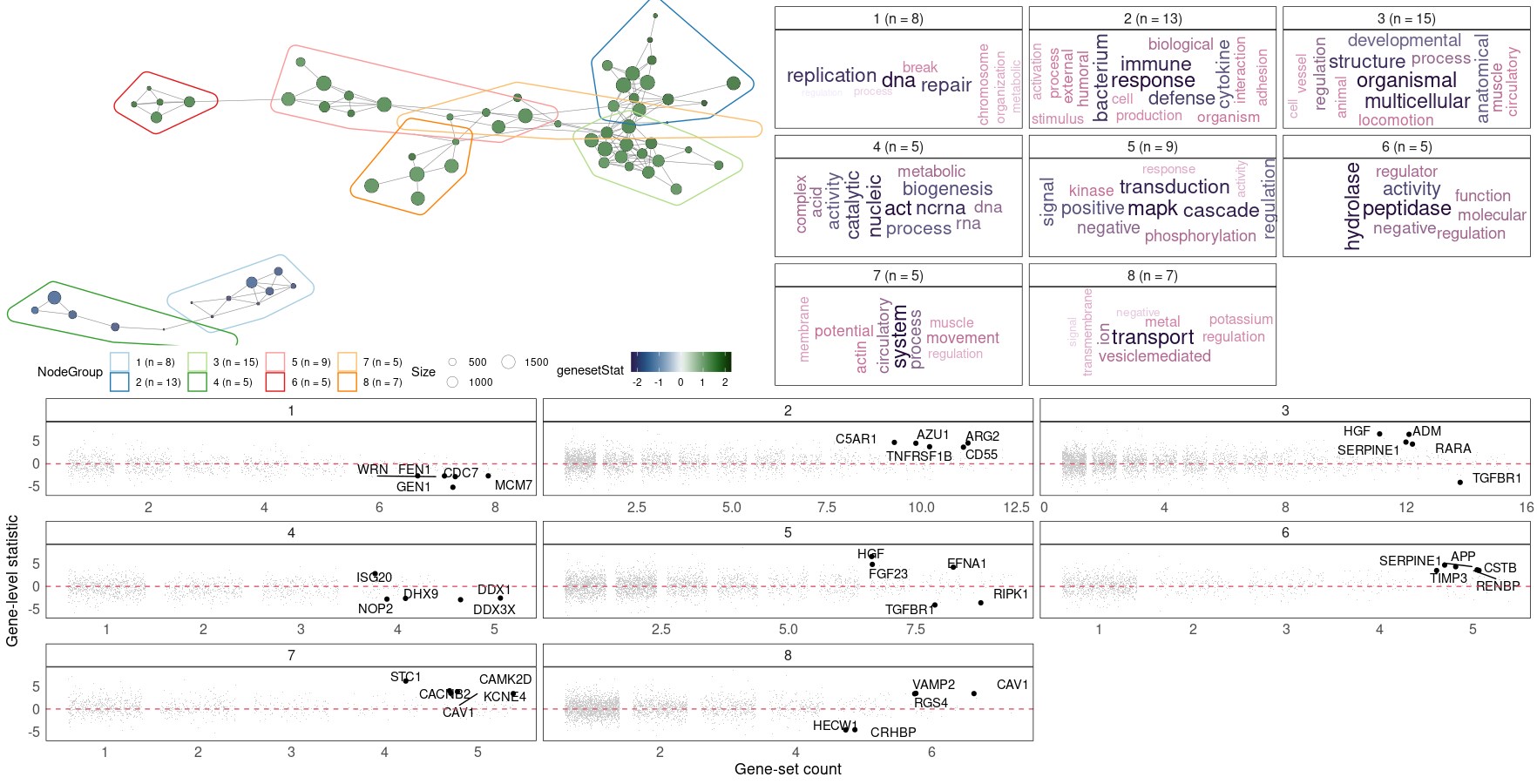

Supplement: SupplementalFig5_ddag005 [file supplementalfig5_ddag005.jpeg]

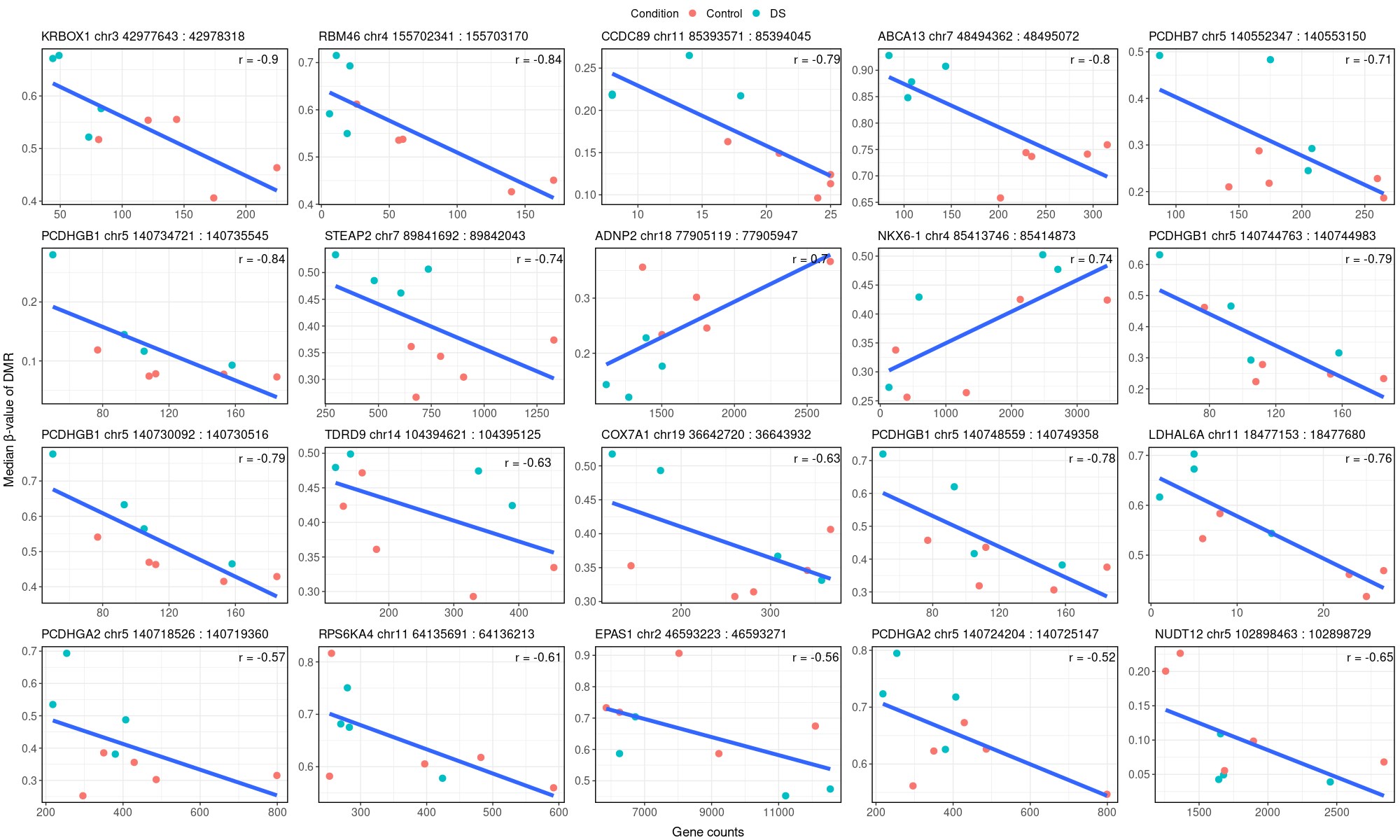

Supplement: SupplementalFig6_ddag005 [file supplementalfig6_ddag005.jpeg]
