# Supplementary material for: Multi-omics investigation of thyroid development and dysfunction in down syndrome
Source: Hum Mol Genet. 2026 Feb 23;35(4):ddag005. doi: 10.1093/hmg/ddag005 (PMC13036834; doi:10.1093/hmg/ddag005)
Supplement: LaufferSupplementaryData_ddag005 [file lauffersupplementarydata_ddag005.docx]

**Supplementary Figures**

**Supplementary Figure 1. Outlier sample detection**

Upper panel: a PCA analysis revealed that samples DS4 and DS5 are clear outliers as opposed to PC1, which explains 87% of variance.

Lower panel: Y-axes represent normalized gene counts. Each sample is represented by a black dot. Panels A, B, D and E visualize gene counts of thyroid marker genes *TPO*, *TG*, *TSHR* and *SLC5A5*. Panels C and F show gene counts of skeletal muscle marker genes *MYH1* and *MYOD1*. Based on gene counts of samples DS4 and DS5, these samples likely mainly consist of skeletal muscle tissue.

**Supplementary Figure 2. Robust principal component analysis (rPCA) results**

Using the grid search algorithm, sparse robust principal components are calculated for each sample, which has proven to be an accurate method for outlier detection in RNA-seq data (36). Sample C3 emerged as a significant outlier from the rPCA.

**Supplementary Figure 3. Histological findings in DS fetal thyroid tissue**

Comparison of DS fetal thyroid tissue with fetal thyroid tissue of controls. Immunolabeling of the images shown was performed with TTF1. The sections show smaller follicle size in the central region and a more heterogeneous appearance of the tissue in DS fetal thyroid tissue compared to non-DS/healthy thyroid tissue.

**Supplementary Figure 4. Normalized counts of selected thyroid-related genes**

Gene counts are shown for 30 selected genes of a thyroid-related gene panel (13), including genes implicated in thyroid gland development and thyroid hormone production, action, and metabolism. Each sample is represented by a black dot.

**Supplementary Figure 5. Clusters of enriched gene sets**

**(A)** Eight clusters of significantly enriched gene sets are given. Gene sets are indicated by circles, colored according to normalized enrichment score. **(B)** Detailed exploration of the clusters by semantic summary of gene set names, of gene sets included in each cluster. **(C)** Genes included in the clusters are indicated with grey dots. X-axes represents occurrence of individual genes in gene sets (included in the cluster), and y-axes represent Wald statistics of genes. The top five genes are given for each cluster.

**Supplementary Figure 6. Significant methylation–expression associations**

Graphs of the 20 significant methylation–expression associations. X-axes represent gene counts, y-axes represent median β-values of the associated DMR. Each sample is represented by a dot. Relationships between DMR methylation and gene expression are given by linear correlation lines (thick blue line), for which Pearson’s correlation coefficient is also given.

**Supplementary Tables**

**Supplementary Table 1. Samples used in final RNA-seq and DNAm models**

**Supplementary Table 2. Bulk RNA-seq analysis results table**

**Supplementary Table 3. Thyroid-related gene set results table**

**Supplementary Table 4. GSEA results tables**

**Supplementary Table 5. DNAm analysis results table**

**Supplementary Table 6. DMR analysis results table**

**Supplementary Table 7. Overlap of RNA-seq and DMR results**

**Supplementary Table 8. eQTM analysis results table**
